# Hypoxia Inducible Factor 3α Plays a Critical Role in Alveolarization and Distal Epithelial Cell Differentiation during Mouse Lung Development

**DOI:** 10.1371/journal.pone.0057695

**Published:** 2013-02-25

**Authors:** Yadi Huang, Joshua Kapere Ochieng, Marjon Buscop-van Kempen, Anne Boerema-de Munck, Sigrid Swagemakers, Wilfred van IJcken, Frank Grosveld, Dick Tibboel, Robbert J. Rottier

**Affiliations:** 1 Department of Pediatric Surgery, Erasmus MC-Sophia Children’s Hospital, Rotterdam, The Netherlands; 2 Department of Bioinformatics, Erasmus MC, Rotterdam, The Netherlands; 3 Department of Genetics, Erasmus MC, Rotterdam, The Netherlands; 4 Department of Biomics, Erasmus MC, Rotterdam, The Netherlands; 5 Department of Cell Biology, Erasmus MC, Rotterdam, The Netherlands; Helmholtz Zentrum München/Ludwig-Maximilians-University Munich, Germany

## Abstract

Lung development occurs under relative hypoxia and the most important oxygen-sensitive response pathway is driven by Hypoxia Inducible Factors (HIF). HIFs are heterodimeric transcription factors of an oxygen-sensitive subunit, HIFα, and a constitutively expressed subunit, HIF1β. HIF1α and HIF2α, encoded by two separate genes, contribute to the activation of hypoxia inducible genes. A third HIFα gene, *HIF3α*, is subject to alternative promoter usage and splicing, leading to three major isoforms, HIF3α, NEPAS and IPAS. HIF3α gene products add to the complexity of the hypoxia response as they function as dominant negative inhibitors (IPAS) or weak transcriptional activators (HIF3α/NEPAS). Previously, we and others have shown the importance of the Hif1α and Hif2α factors in lung development, and here we investigated the role of Hif3α during pulmonary development. Therefore, HIF3α was conditionally expressed in airway epithelial cells during gestation and although HIF3α transgenic mice were born alive and appeared normal, their lungs showed clear abnormalities, including a post-pseudoglandular branching defect and a decreased number of alveoli. The HIF3α expressing lungs displayed reduced numbers of Clara cells, alveolar epithelial type I and type II cells. As a result of HIF3α expression, the level of Hif2α was reduced, but that of Hif1α was not affected. Two regulatory genes, Rarβ, involved in alveologenesis, and Foxp2, a transcriptional repressor of the Clara cell specific Ccsp gene, were significantly upregulated in the HIF3α expressing lungs. In addition, aberrant basal cells were observed distally as determined by the expression of Sox2 and p63. We show that Hif3α binds a conserved HRE site in the Sox2 promoter and weakly transactivated a reporter construct containing the Sox2 promoter region. Moreover, Hif3α affected the expression of genes not typically involved in the hypoxia response, providing evidence for a novel function of Hif3α beyond the hypoxia response.

## Introduction

The lung originates from the primitive foregut early in the development of land dwelling organisms, and through a complex interplay of signaling molecules the future airway epithelium and surrounding mesenchyme develop into the highly structured arbor-like bronchial-vascular tree (reviewed in [1], [2], [3]). Normal development in mammals occurs in a relative hypoxic environment, which is beneficial for lung organogenesis [4], [5]. Cellular responses to different levels of oxygen are important for development and homeostasis [6], and the most important oxygen-sensing mechanism to protect cells from oxygen toxicity is the transcriptional response mediated by Hypoxia Inducible Factors (HIF), which are also expressed in the lungs [7].

HIFs are critical mediators of the hypoxic cellular response and regulate cellular adaptation by transactivating genes involved in angiogenesis, metabolism and cellular homeostasis (for recent reviews see [6], [8], [9]). HIFs are heterodimeric transcription factors which have two structurally related subunits, an oxygen sensitive HIFα subunit and a constitutively expressed HIFß or ARNT subunit (Aryl hydrocarbon Receptor Nuclear Translocator). Both subunits belong to the transcription factor family containing a basic Helix-Loop-Helix (bHLH) and a Per/ARNT/Sim (PAS) domain at the N-terminus, which mediate heterodimerization and DNA binding [10], [11]. HIFß is expressed ubiquitously and as such, the level and expression patterns of the HIFα proteins are mostly determining the activity of the heterodimers [12]. Currently, three genes have been identified in human and mouse that encode HIFα isoforms, *HIF1α* [10], [11], *HIF2α* or *EPAS1* [13], [14], [15], and *HIF3α* [16], [17], [18], [19], [20]. Aside from the N-terminal bHLH/PAS domain, the HIFα subunits contain an Oxygen-Dependent Degradation Domain (ODDD) in the center of the protein, an N-terminal transactivation domain (NTAD) and a C-terminal transactivation domain (CTAD) [21], [22], [23], [24], [25]. The CTAD is absent in the HIF3α subunit, which significantly reduces the transcriptional activity of the protein [16], [26]. The three α subunits are post-transcriptionally regulated by prolyl hydroxylase domain-containing enzymes (PHD1-3), which hydroxylate with different specificity the HIFα subunits at two critical prolyl residues in the ODDD under normoxic conditions [22], [27]. The PHD proteins are dioxygenases which require oxygen for their function and as such are sensitive to oxygen concentrations, losing their activity under low oxygen concentration [22]. The hydroxylated HIFα proteins are poly-ubiquitinylated and targeted for 26S proteosomal degradation through the von Hippel-Lindau (pVHL)/Elongin BC/Cul2 ubiquitin-ligase complex [28], [29], [30], [31], [32], [33], [34]. Under low oxygen conditions, the PHD proteins are inactive, so the HIFα proteins are not hydroxylated and stable. They will translocate to the nucleus and dimerize with HIF1β, leading to the transcription of target genes, such as EPO and VEGF, through the binding to specific DNA seqences (Hypoxia Responsive Elements, HRE) [8], [35], [36]. Aside from the regulation of the stability of the HIFα isoforms by PHDs, additional regulatory activities are identified. The oxygen-dependent asparaginyl hydroxylase Factor Inhibiting HIF (FIH), a member of the Fe(II) and 2-oxoglutarate-dependent dioxygenase, hydroxylates a conserved asparaginyl residue in the CTAD, preventing the association of HIFα with the p300 coactivator [37], [38], [39]. In addition to these hydroxylation dependent regulation of HIFα isoforms, several other posttranslational modifications have been identified (for review, see [8], [40], [41]).

The regulation and functions of the HIF3α gene and isoforms is very complex, contrasting HIF1α and HIF2α. The *HIF3α* locus gives rise to different splice variants, resulting in three protein isoforms, HIF3α, NEPAS (neonatal and embryonic PAS) and IPAS (inhibitory PAS) [19], [20], [42]. HIF3α and NEPAS only differ in the first eight N-terminal amino acids due to alternative exon usage. IPAS and NEPAS are hypoxia inducible, whereas HIF3α is not because of alternative usage of promoters [43], [44]. HIF3α expression is induced under hypoxia in several organs, including cortex, hippocampus, lung, heart, kidney, cerebral cortex [17], [45], [46]. NEPAS is almost exclusively expressed during late embryonic and neonatal stages of development, especially in the lung and heart, while HIF3α mRNA is rarely detectable during embryonic and neonatal stages [42]. HIF3α has a high homology to HIF1α and HIF2α at the N-terminus, but only a low degree of sequence similarity across the C-terminus [26]. The HIF3α/HIF1ß (HIF3) and NEPAS/HIF1ß dimers suppress basal and hypoxia induced reporter gene activation, as well as HIF1 (HIF1α/HIF1ß) or HIF2 (HIF2α/HIF1ß) driven expression [16], [42]. HIF3 binds to HRE sites in promoter regions, but the transcriptional activity is much weaker than that of HIF1 and HIF2, because it lacks the CTAD [16], [26], [42]. Therefore, both HIF3α and NEPAS serve as competitors of HIF1 and HIF2 dependent transcription, not only by occupying identical promoter regions, but also by associating with the same HIF1ß partner [16], [42]. The splice variant IPAS lacks both the NTAD and CTAD domains producing a dominant negative regulator of the HIF1α and HIF2α dependent pathway [16], [18], [43]. It was shown that IPAS directly associates with HIFα isoforms, thereby displacing Hif1β, and the resulting IPAS/Hifα dimer is unable to bind to DNA [18]. Both short HIF3α isoforms related to IPAS in human and the IPAS in mouse have antagonistic effects on the expression of HIF1 and HIF2 dependent hypoxia regulated target genes [47]. Thus, the *HIF3α* locus encodes isoforms generally thought to act as negative regulators of the hypoxic response.

The importance of the hypoxia response was shown by the identification of mutations in the VHL-HIF pathway in different human diseases (reviewed in [9]). Specific gene ablation studies in mice also added to the knowledge on the pleiotropic effects of the members of the hypoxia response pathway. Complete ablation of this pathway through inactivation of Hif1ß resulted in a severe lethal phenotype with defective angiogenesis of the yolk sac and branchial arches, stunted development and embryo wasting [48], [49]. Hif1α knockout mice also died early during development with cardiac malformations and vascular defects [50]. Hif2α null mice displayed a pleiotropic phenotype ranging from premature death until postnatal abnormalities, depending on the background of the mouse strain [51], [52], [53], [54]. The neonates that survived suffered from breathing problems and did not produce sufficient surfactant phospholipids and surfactant associated proteins [51]. It is interesting to note that the inactivation and ectopic activation of Hif2α showed comparable phenotypes, suggesting that type II cells require different levels of Hif2α at distinct phases of type II cell maturation [51], [55]. Homozygous mutant NEPAS/Hif3α^-/-^ mice were alive at birth, but displayed enlarged right ventricle and impaired lung remodelling, suggesting that NEPAS/Hif3α is important in lung and heart development during embryonic and neonatal stages [42]. Interestingly, the *Hif3α* gene contains hypoxia response elements in its promoter region and has been shown to be a transcriptional target of Hif1α [56].

In order to understand the precise role of Hif3α during pulmonary epithelium development, we generated transgenic mice with an inducible *HIF3α* gene. Mice expressing the *HIF3α* transgene in the developing airways showed a post-pseudoglandular branching defect with a reduced number of airspaces and a clear reduction in the number of alveolar type I and type II cells. Importantly, expression of the HIF3α transgene did not lead to changes in the levels of Hif1α, but affected Hif2α. The lungs of the HIF3α expressing mice showed an upregulation of genes normally expressed in the proximal parts of the lung, while genes only expressed in distal parts of the lung were downregulated. Specifically, Foxp2, a repressor of distal cell markers, and Rarß were induced in the lungs of Hif3α expressing mice, which may explain the reduction in the number of distal cell types. Furthermore, we showed that Hif3α binds a conserved HRE in the Sox2 promoter and induces the expression of a Sox2 promoter driven reporter gene, explaining the appearance of aberrant Sox2- and p63 positive cells. Collectively, our results show that Hif3α is involved in modulating correct development of the lung epithelium.

## Materials and Methods

### Generation of transgenic animal

The myc epitope encoding sequence (EQKLISEEDL) was cloned directly after the endogenous ATG start codon of the full length human HIF3α cDNA (GenBank: BC080551) and subcloned into a modified pTRE-Tight vector [55]. Transgenic lines were produced by pronuclear injection of FVB/N fertilized eggs, and tail tip DNA of transgenic lines was initially genotyped by Southern blot analysis, after which positive lines were routinely checked by PCR, using transgene-specific primers (sense: 5′-GTCAAGCTTATGGCGCTGGGGCTGCAGCG; antisense 5′- GCATCTAGATCAGTCAGCCTGGGCTGAGC). Three independent lines were initially analyzed, which all produced the same phenotype as described in this manuscript. Lung-specific expression of the HIF3α transgene, i-Tg-mycHIF3α, was obtained by crossing the mycHIF3α lines with the SPC-rtTA transgenic mice (A generous gift of Jeffrey Whitsett). Administration of doxycycline to pregnant mothers from gestational age 6.5 onward in the drinking water (2 mg/ml, 5% sucrose) resulted in lung epithelium-specific expression. Each experiment was performed with at least three independent litters containing double transgenic, single transgenic and wild type pups. All double transgenic animals receiving doxycycline expressed mycHIF3α in the pulmonary epithelium and showed the described phenotype. Mice were housed under standard conditions at 40–50% relative humidity and 21±1°C (12/12 hour dark/light cycle) with food and water ad libitum. All animal experiments were performed according to the Dutch and European guidelines and approved by the local ethics committee (DEC Nr 1657, 1833 and 2206).

### Immunohistochemistry

Immunohistochemistry was essentially performed as previously described [57]. Briefly, lungs were dissected and fixed in formal saline (BDH) overnight at 4°C before processing for paraffin embedding according to routine protocols Antigen retrieval was performed with microwave treatment in 10 mM citric acid buffer pH 6.0 or Tris-EDTA. Sections were blocked with 5% BSA or 5% ELK in PBS for 10min and incubated with primary antibody diluted in 5% BSA or 5% ELK in PBS overnight at 4. The following antibodies were used: Myc (9E10, Roche; 4A6, Millipore), Hif3α (Ab2165, Abcam; NBP1-03155, Novus Biologicals), ß-tubulin IV (bioGenex), proSP-C (Chemicon), T1α (University of Iowa Hybridoma bank), Ttf1 (Thermo), Ccsp (seven hills), Sox2 (seven hills), Foxp2 (Abcam), Lpcat1 (Seven hills Bioreagents), α-Sma (Thermo), Ki67, cGRP Secondary antibodies against the correct IgG species were conjugated with peroxidase (Dako).

Lungs were imaged using an Olympus BX41 microscope and DP71 camera (Olympus, Zoeterwoude, The Netherlands). Subsequent airspaces counting were performed with SIS Softward Cell D (Olympus). Three independent samples of control and double-transgenic lungs of gestational age E18.5 were used to count the number of airspaces on a selected surface area (140000 µm^2^) on those selected lung samples.

### Microarray analysis

Lungs of three control and three double transgenic embryos were dissected at E18.5 and the middle and caudal lobes were used for total RNA isolation with Trizol reagent according to the manufacturer’s instructions (Invitrogen life technologies, Carlsbad, CA, USA). RNA was purified using the RNeasy MinElute Cleanup kit. (Qiagen, Valencia, CA, USA) and cDNA was synthesized from 3 µg RNA using the GeneChip Expression 3′-Amplification Reagents One-Cycle cDNA Synthesis kit (Affymetrix, Santa Clara, CA, USA). Biotin-labelled cRNA synthesis, purification and fragmentation were performed according to standard conditions. Fragmented biotinylated cRNA was subsequently hybridized onto Affymetrix Mouse Genome 430 2.0 microarray chips. After normalization, the data were analysed with OmniViz software, version 3.6.0 (Omniviz, Inc., Maynard, MA, USA).

Functional annotation of the statistical analysis of microarrays results was done using Ingenuity Pathway Analysis (Ingenuity, Mountain View, CA) and DAVID (http://david.abcc.ncifcrf.gov). The results are shown for biological processes, which are significantly (P <0.05) enriched after multiple testing.

### RT-PCR

RNA isolation and subsequent quantitative PCR analysis was essentially performed as previously described [7]. Gene-specific primer sets were Abca3: 5′-TTACGGTCCAAGTTCCTGAG-3′ and 5′-TAACATCAGCACCTTAGAGCC-3′; Aqp5: 5′-GTGGTCATGAATCGGTTCAG-3′ and 5′-CAAGTAGAAGTAGAGGATTGCAG-3′; Epas1: 5′-CTGTGACGACAGAATCTTGG-3′ and 5′-GGCATGGTAGAACTCATAGG-3′; Foxp2: 5′-TGTCATCAGAGATTGCCC-3′ and 5′-ATAGCCTGCCTTATGAGTG-3′; Rarß: 5′-AACTGCGTCATTAACAAGGTC-3′ and 5′-TCATTCCTAACAGACTCTTTGG-3′; Scd1: 5′-GAGCCACAGAACTTACAAGG-3′ and 5′-GTACACGTCATTCTGGAACG-3′; Sftpd: 5′-GGAAGCAATCTGACATGCTG-3′ and 5′-GAGGCTCTTCATTTCTGCTC-3′. Standard deviations of the duplicates are calculated with the SPSS program (Independent-samples T test), which also generated the P values.

### Luciferase reporter activity assays

HEK293T cells were transfected in duplo with Lipofectamine 2000 (Invitrogen) with a total concentration of 500 ng DNA/well, using 9*HREluc (Gift from Manuel Landazuri), pGL3-mpSox2 and pGL3-mpSox2delta (Named Sox2-Luc and ΔSox2-Luc; Gift from Victoria Moreno), Hif2α-pcDNA3, (gift from Carole Peyssonnaux), Hif3α-pcDNA3 or pcDNA3. Cells were lysed with passive lysis buffer (Promega) 24-hours after transfection and processed for lucifease analysis by the addition of the LARII reagent (Promega), which was subsequently quantified with the VICTOR luminometer. A construct containing the renilla gene (10 ng/well) was co-transfected in each well to serve as an internal control for transfection efficiency. The renila luciferase activity was quantified by addition of Stop&Glio reagent and also detected with the VICTOR luminometer. The experiment was repeated three times, and all samples were measured at least in duplo. The average luciferase activity was calculated and divided by the average of renilla activity. Standard deviations were measured with the SPSS program (Independent-samples T test), which also generated the P values.

### Chromatin immunoprecipitation (ChIP)

ChIP assay was performed essentially as previously described [58], with some modifications. Chromatin-protein complexes of confluent A549 cells were fixed by adding 1% formaldehyde to the cultures. Nuclear extracts were made and chromosomal DNA was fragmented by sonication. Equal amounts of DNA was diluted 1:10 with ChIP dilution Buffer (0.01% SDS, 1.1% Triton X-100, 1.2 mM EDTA, 16.7 mM Tris-HCl pH 8.1 and 167 mM NaCl) and the samples were pre-cleared with 80 µl prot A/G agarose beads for 1 hour, after which the sample was split in equal volumes and incubated O/N with 6 µg antibody specific for HIF3α (NBP1-03155) or control IgG (rabbit). Immune complexes were subsequently purified by adding 80 µl of prot A/G beads, which were washed several times before the immune-precipitated DNA was eluted with elution buffer (1% SDS and 0.1 m NaHCO_3_). After de-crosslinking the DNA-protein complexes by incubation at 65°C O/N with 200 mM NaCl, the eluted DNA was phenol-extracted, precipitated and qPCRs were performed to analyze the enrichment of HIF3α specific binding to the HRE in the SOX2 gene using the following primer set 5′-CAAGTGCATTTTAGCCACAAAG-3′ and 5′-CCCAAGAGGGTAATTTTAGCCG-3′, while the primers for the ARRDC3 and EGLN3-D were described previously [36], [59]. The data are the average of two independent ChIP assays, which were each analyzed by duplicate qPCRs, and are represented as the fold enrichment of the specific immune precipitation compared to the control IgG precipitation.

## Results

### Ectopic expression of mycHIF3α causes late branching defects

**Figure 1:**
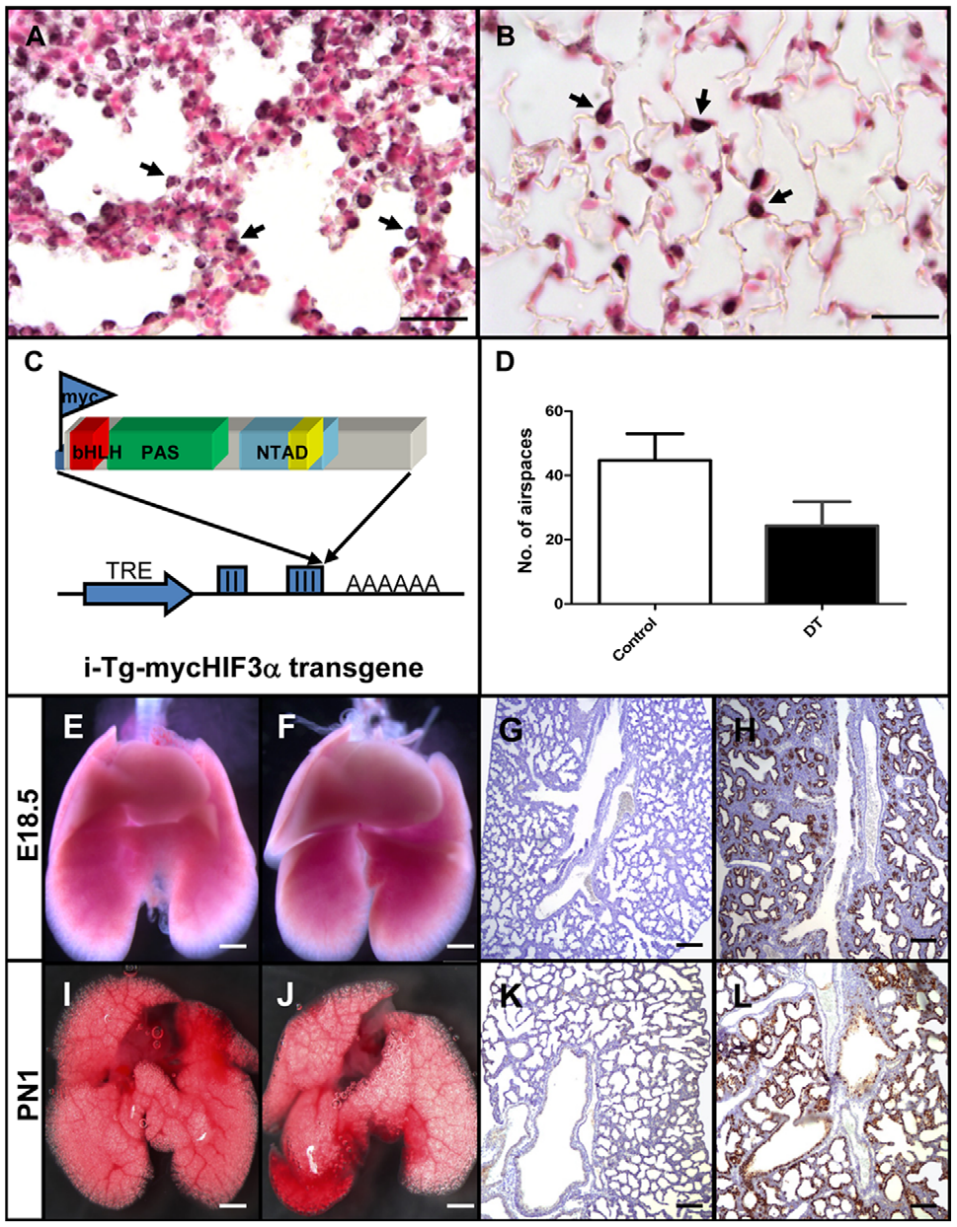
Enhanced expression of HIF3α results in late branching defect. Endogenous expression of Hif3α was detected in epithelial cells at gestational age E18.5 (A, arrows) and in type II pneumocytes in adult mice (B, arrows). (C) Graphic representation of the tet-inducible Hif3α/NEPAS cDNA construct used to generate transgenic mice. TRE is the Tet-responsive element containing minimal promoter, II and III refer to exon 2 and 3 of the ß-blobin gene and AAAAAA is the poly-adenylation signal. Indicated are the position of the myc-epitope, and the bHLH, PAS and NTAD domains (see text) (D) Quantification of the number of airspaces in the lung. Three independent samples of control and double-transgenic lungs at gestational age E18,5 were used to count the number of airspaces. External appearances of control (E, I) and double transgenic mycHIF3α (F, J) lungs at E18.5 days of gestation (E18.5) and post natal age 1 (PN1) do not show apparent differences. Histological analysis of control (G, K) and double transgenic lungs (H, L) showed decreased number of alveolar spaces and reduced branching in the double transgenic lungs (H and L). Anti-Myc epitope staining confirmed the expression of the mycHIF3α transgene in double transgenic lungs (H and L), which is absent in control lungs (G and K). Scale bars: Scale bars: 25 µm (A, B), 2 mm (E, F, I, J) or 200 µm (G, H, K, L).

Previously, it was shown that homozygous NEPAS/Hif3α knockout mice were viable, but displayed an enlarged right ventricle and impaired lung remodelling, suggesting that Hif3α plays an important role during pulmonary development. However, the precise role of Hif3α during the formation of the lung is not fully understood. We first analyzed the endogenous expression of Hif3α in normal fetal lungs isolated at the end of gestation (E18.5) and in lungs of adult mice (8 weeks). Hif3α positive cells were present in the epithelium of the developing lung, as well as in the type II pneumocytes of the adult lung (arrows in Figure 1A, B). In order to determine the precise role of Hif3α in the epithelium during lung development, and more specifically in type II pneumocytes, we generated transgenic mice carrying a myc-epitope tagged HIF3α under the control of a doxycycline-inducible tet-on promoter (i-Tg-mycHif3α; Figure 1C). Expression of mycHIF3α in embryonic lung epithelium was established by crossing the i-Tg-mycHIF3α transgenic line with the established SPC-rtTA line, which drives the expression of the rtTA gene in epithelial cells of the embryonic lung [60]. Pregnant females from timed matings between SPC-rtTA and i-Tg-mycHIF3α mice received doxycycline to induce the expression of the HIF3α transgene in double-transgenic fetuses. Lungs isolated from doxycycline-induced or non-induced single i-Tg-mycHIF3α or SPC-rtTA transgenic mice, or lungs from non-induced double transgenic i-Tg-mycHIF3α/SPC-rtTA animals appeared indistinguishable from wild type lungs. Doxycycline-induced, double-transgenic pups were born at Mendelian ratio and did not show obvious external abnormalities compared to their control litter mates.

In order to determine whether expression of mycHIF3α leads to pulmonary development defects, we analyzed lungs of double-transgenic animals and control lungs at different gestational ages. Macroscopic analysis of isolated lungs did not show clear abnormalities in double-transgenic animals at gestational ages E16.5, E17.5, E18.5 days and postnatal day 1 (PN1) (Figures 1E and F, I and J; Figure S1). Histological examinations at E16.5 did not show clear differences between control and mycHIF3α transgenic lungs (Figures S1C and D). However analysis of a series of developmental ages clearly showed aberrant alveolar airspaces in mycHIF3α expressing lungs starting at E17.5 compared to controls (Figure S1G, H). mycHIF3α expressing lungs contained significant fewer alveolar spaces compared to control ones at E18.5 and PN1 (Figures 1D). Staining with a specific antibody against the myc-epitope confirmed the expression of transgenic mycHIF3α protein in the epithelium of double-transgenic lungs (Figures 1H and L, Figure S1). The abnormal alveolar spaces remain present in the PN1 stages, but apparently, the mice do not suffer from respiratory distress, indicating that the initial requirements for life are present. So, we conclude that mycHIFα expression in epithelial cells leads to aberrant alveolar formation and affects late branching morphogenesis during pulmonary development.

**Figure 2:**
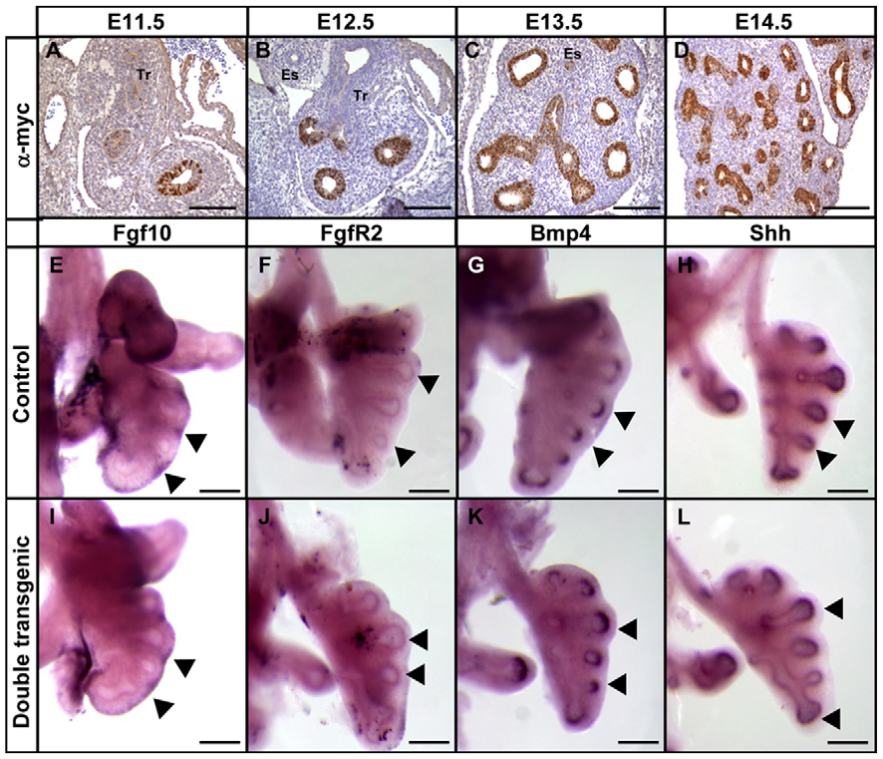
Expression of genes involved in branching morphogenesis. Analysis of the distribution of mycHIF3α early in lung development in double transgenic animals at E11.5 (A), E12.5 (B), E13.5 (C) and E14.5 (D). Whole mount in situ hybridization to detect the expression and localization of Fgf10 (E and I), FgfR2 (F and J), Bmp4 (G and K) and Shh (H and L) in lungs isolated at gestational age E12.5 from control (E–H) and mycHIF3α double transgenic animals (I–L). Tr: Trachea; Es: Esophagus. Scale bars: 200 µm.

This post-pseudoglandular branching defect prompted us to analyze the expression of the mycHIF3a at early embryonic stages of development. This showed that the transgene is expressed in a non-uniform manner in the epithelium of early E11.5 lungs (Figure 2A), but gradually all epithelial cells express the transgene (Figure 2B-D). Next, we analyzed whether the primary airway branches appropriately expressed some of the major branch-inducing genes [2]. Therefore, embryonic lungs of controls and double transgenic animals were isolated at gestational age 12.5. At this stage of development, the primary bronchi are already present, and these branches start to form secondary and tertiary branches. The expression of *Fgf10*, the growth factor with a very potent branch-inducing activity, was found in the mesenchymal compartment, alongside the epithelium that is in the process of branching (Figure 2E and I, arrows). Moreover, its receptor, *FgfR2*, was detected at the tips of the epithelium, in close proximity of the Fgf10 signal (Figure 2F and J). Next, we also analyzed the expression of two genes known to be induced as a result of the Fgf10-FgfR2 signalling, *Shh* and *Bmp4*. Both genes were also expressed in the epithelium at the same location as the FgfR2, indicating that the Fgf10-FgfR2 signalling cascade is intact (Figure 2G and K; H and L). In addition, quantitative PCR analysis of embryonic lungs isolated at E12.5, E15.5 and E17.5 of controls and double transgenic mice using primers specific for FgfR2, FgfR2-IIIb, FgfR2-IIIc, Bmp4 and Spry did confirm the absence of differential expression of these important branch-inducing genes (data not shown). In conclusion, no differences in expression pattern were observed for the early branch-inducing genes between controls and double transgenics, suggesting that the initiation of the branching process occurred normally.

### mycHIF3α expression inhibits Clara cells differentiation

**Figure 3:**
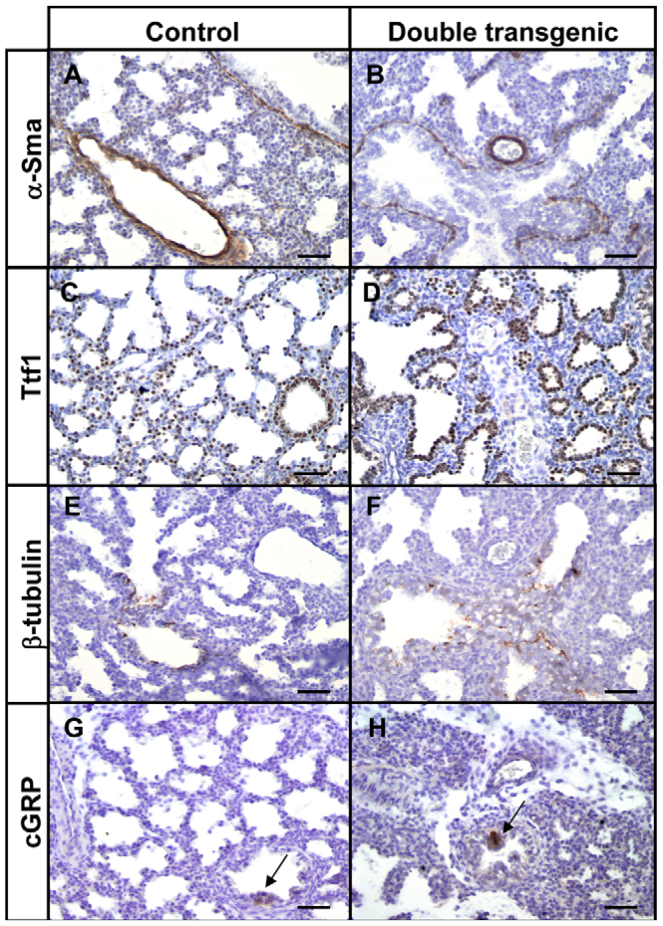
Normal differentiation of proximal epithelial cells in mycHIF3α transgenic lungs. The site and expression pattern of α-Sma (A and B), Ttf1 (C and D), β-tubulin (E and F) and cGRP (arrows in G and H) are comparable between control and mycHIF3α double transgenic lungs at gestational age E18.5. Scale bars: 100 µm.

**Figure 4:**
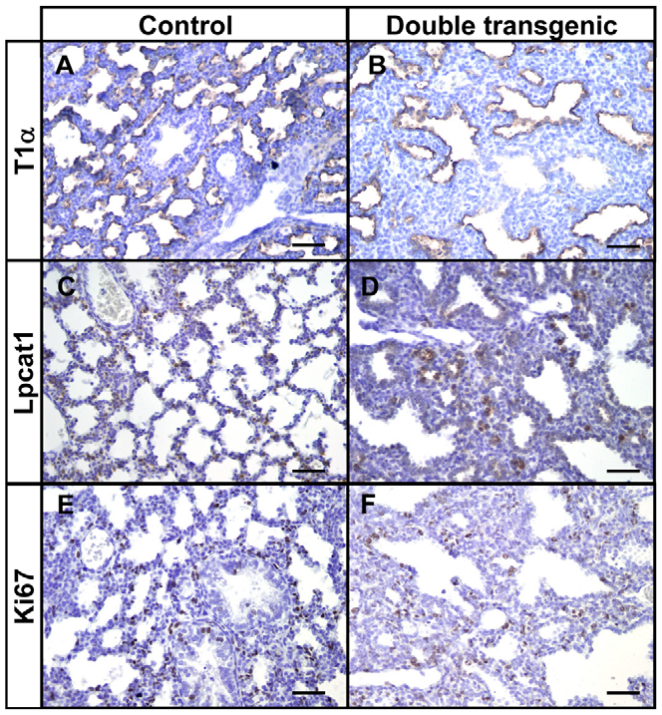
Normal differentiation of distal epithelial cells in mycHIF3α transgenic lungs. The site and expression pattern of T1α (A and B), Lpcat1 (C and D) and Ki67 (E and F) are comparable between control and mycHIF3α double transgenic lungs at gestational age E18.5. Scale bars: 100 µm

Since we observed significant alveolar changes and aberrant branching morphogenesis, we analyzed the integrity and differentiation potential of fetal transgenic lungs by immunohistochemistry with cell-specific markers. The smooth muscle cell component of the mesenchyme (α-Sma) did not reveal striking differences between control and transgenic lungs (Figures 3A, B). Thyroid transcription factor (Ttf1) was expressed in nearly all epithelial cells in both control and transgenic lungs (Figures 3C, D). Ciliated cells (β-tubulin) and neuroendocrine cells (cGRP) were present in proximal conducting airways of control and transgenic lungs at gestational age E18.5 (Figures 3E, F and 3G, H, arrows). Moreover, both type I (T1α; Figure 4A, B) and type II pneumocytes (Lpcat1; Figures 4C, D) were present in the alveolar regions. These results indicate that differentiation into the various epithelial cell types is not hampered by Hif3α, although the total number of each cell type may be different. In addition, no differences were observed in the proliferation of epithelial or mesenchymal cells between control and transgenic lungs as indicated by Ki67 staining (Figure 4E, F).

**Figure 5:**
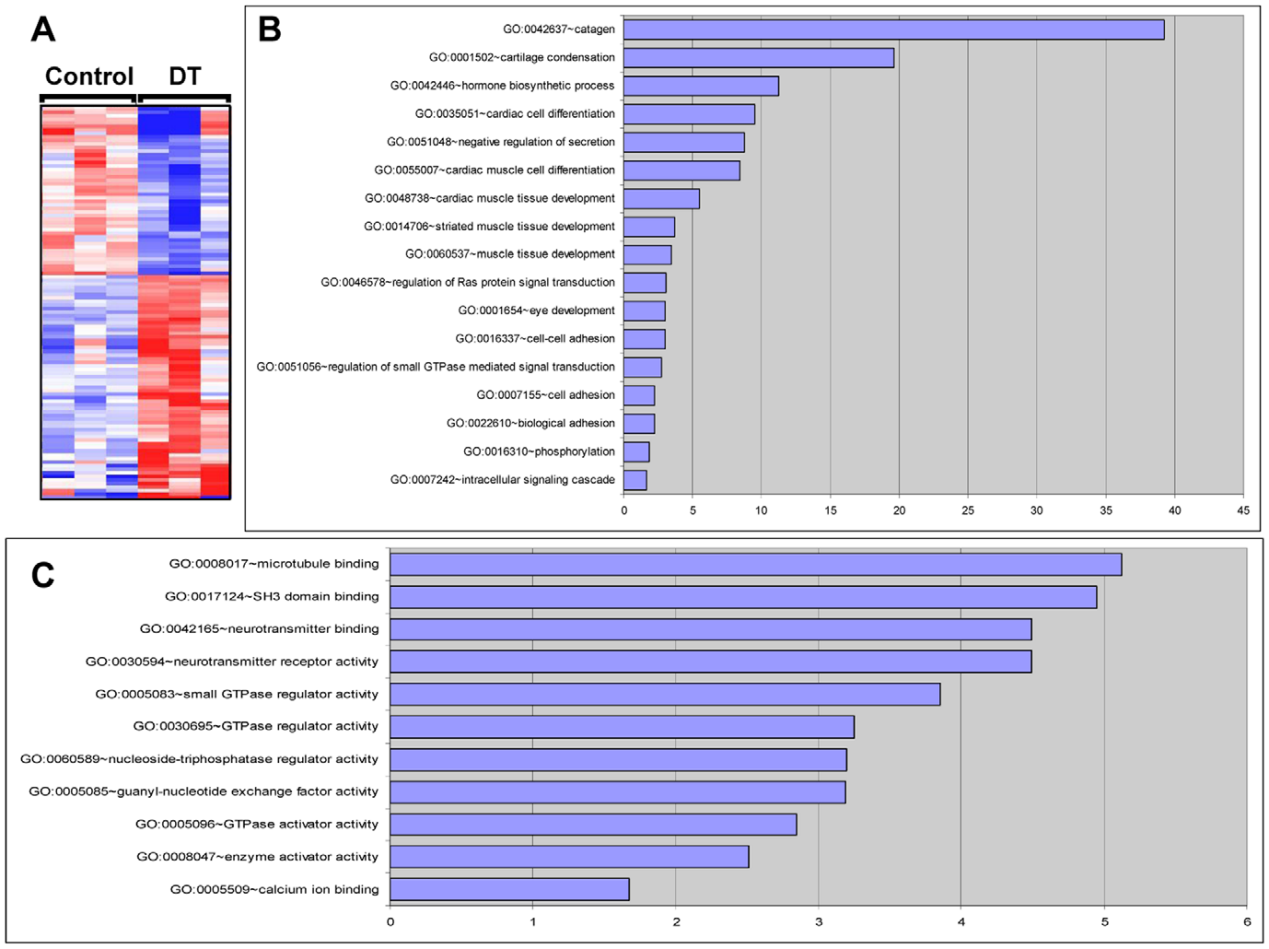
Transcriptome analysis of mycHIF3α expressing lungs. Treescape showing that the transcriptome of the lungs of the mycHIF3α expressing animals are significantly different from that of the control lungs (A). The red color indicates the upregulated genes and the blue color indicates downregulated genes. The expression of the genes presented in the treescape is at least 1.5 fold changed with a false discovery rate (FDR) of 10%. The top 10 biological processes (B) and molecular functions (C) of the differentially expressed genes are shown.

**Figure 6:**
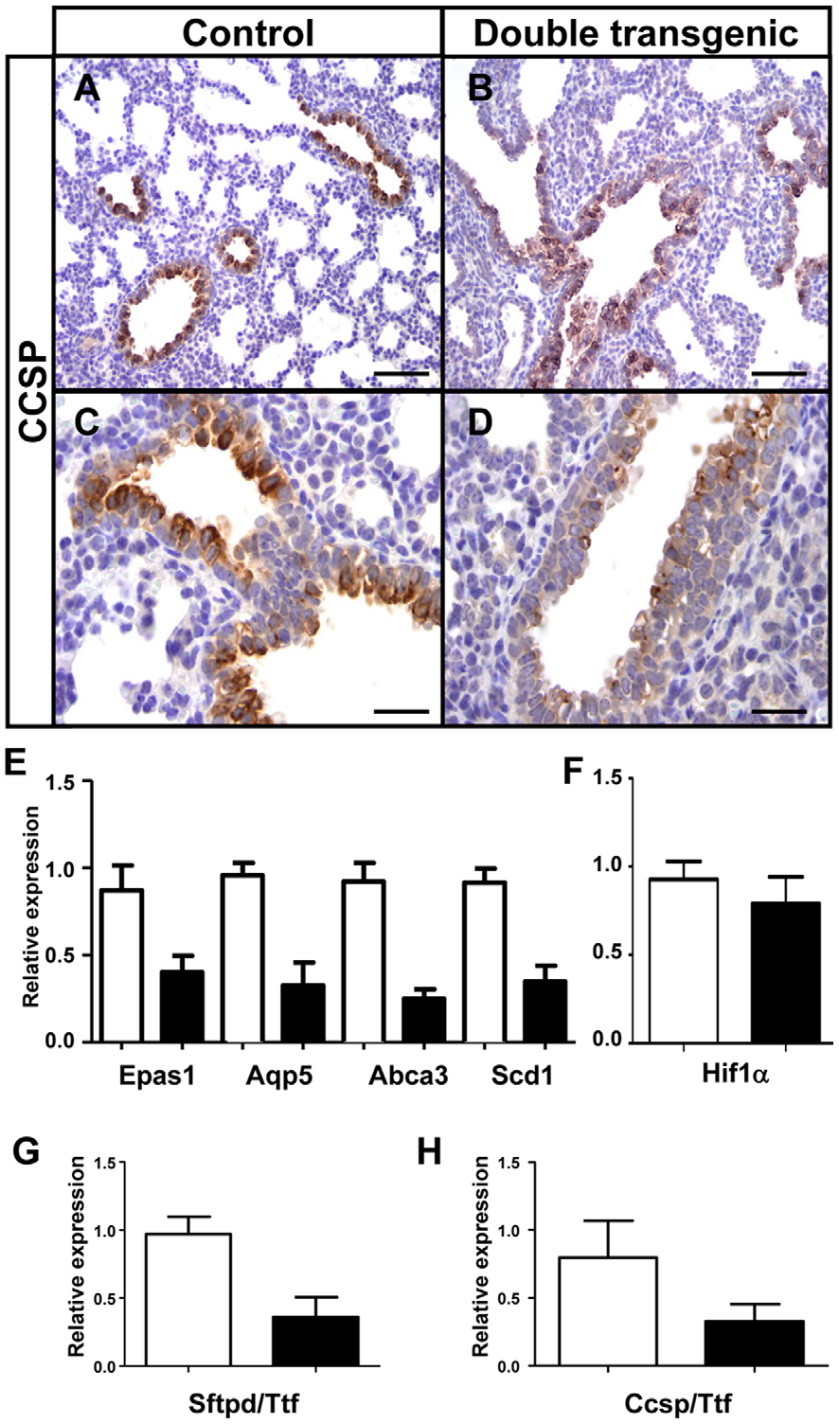
mycHIF3α reduces the number of Clara cells. The expression of the Clara cell marker, Ccsp, was strongly decreased in mycHIF3α transgenic lungs at gestational age E18.5 compared to controls (A and C versus B and D). (E) Alveolar epithelial cell markers are downregulated in Hif3α transgenic lungs at gestational age E18.5 as shown by quantitative PCR. *Epas1* (0,4 + 0.1 versus control 0.87 + 0.1, n = 3 each, P = 0.012), *Aqp5* (0.33+ 0.1 versus control 0.96 + 0.1, n = 3 each, P = 0.005), *Abca3* (0.25 + 0.1 versus control 0.92 + 0.1 n = 3 each, P = 0.002), *Scd1*(0.35 + 0.1 versus control 0.92 + 0.1, n = 3 each, P = 0.001). (F) There is no significant change in the mRNA expression of Hif1α gene (0,8 + 0.1 versus control 0.7 + 0.1, n = 3 each, P>0.05). Quantification of the number of (G) type II pneumocytes (*Sftpd* over *Ttf1*, 0.36 + 0.1 versus control 0.9 + 0.1; n = 3, P = 0.01) and (H) Clara cells (*Ccsp* over *Ttf1*, 0.3 + 0.1 versus control 0.82 + 0.1; n = 5, P = 0.01) showed a significant reduction of in the Hif3α double transgenic animals. White bars represent control lung samples, black bars represent mycHIF3α double transgenic lung samples. Scale bars: 100 µm (A, B) and 50 µm (C,D).

Next, three mycHIF3α-expressing lungs and three control lungs were processed at gestational age 18.5 days for microarray analysis, to elucidate the origin of the aberrant branching morphogenesis. Hierarchical clustering of differentially expressed genes revealed large differences between controls and double transgenic lungs (Figure 5A) and the major biological processes (Figure 5B) and molecular functions (Figure 5C) are indicated. Although mycHIF3α does not prevent the differentiation of epithelial cells into Clara cells, we noticed that the number of Clara cells was significantly reduced. Both in the microarray analysis as well as the qPCR validation showed downregulation of the Ccsp gene in mycHIF3α transgenic mice. These gene expression results were confirmed by immunohistochemistry, showing that Ccsp positive cells were less prominent in the proximal airways of the Hif3α expressing lungs compared to control lungs (Figures 6A-D). Quantification of the total number of Clara cells revealed a significant reduction in the double transgenic mice (Figures 6H). So, our data show that mycHIF3α expression inhibits Clara cells differentiation during pulmonary development.

### mycHIF3α induces airway epithelial cells to differentiate into proximal cell types

Analysis of the microarray data revealed that genes associated with proximal cell types of the lung appeared to be upregulated, whereas genes specifically expressed in distal epithelial cells were downregulated (Table 1 and Table 2). The induction of proximal markers is reflected by the significant downregulation of genes specific for the distal lung epithelium. The type 1 pneumocyte cell marker Aquaporin 5 (*Aqp5*) was dowregulated in the Hif3α expressing mice, as were three genes specifically expressed in type II pneumocytes, stearoyl-coenzyme A desaturase (*Scd1*), surfactant associated protein D (*Sftpd*) and ATP-binding cassette (ABC) subfamily A3 (*Abca3*) (Figure 6E) [61], [62], [63]. Quantification of the number of type II pneumocytes present in the Hif3α expressing lungs using *Sftpd* in reference to *Ttf1* confirmed a significant reduction in these cells (Figure 6G). Since we are inducing the Hif3α family member of hypoxia inducible genes, we analyzed the expression of Hif1α and Hif2α in the transgenic lungs. Although no apparent difference could be detected for Hif1α (Figure 6F), but we did notice a significant downregulation of Hif2α (*Epas1*) (Figure 6E). Previously, we showed that Hif2α is involved in maturation of type II pneumocytes, so the reduction of *Epas1* expression could be directly related to the loss of type II cells.

**Table 1:** Significant upregulated genes in the mycHIF3α expressing lungs.

Gene symbol	Gene name	Entrez ID	Fold Change
Dub2a	deubiquitinating enzyme 2a	384701	6,22
Naaladl2	N-acetylated alpha-linked acidic dipeptidase-like 2	635702	2,16
Cldn6	claudin 6	54419	2,14
Hspa1a	heat shock protein 1A	193740	2,14
Fbn2	fibrillin 2	14119	2
ATP6	ATP synthase F0 subunit 6	17705	1,87
Rimklb	ribosomal modification protein rimK-like family member B	108653	1,83
Sema3e	sema domain, immunoglobulin domain (Ig), short basic domain, secreted, (semaphorin) 3	20349	1,8
Tinag	tubulointerstitial nephritis antigen	26944	1,71
Mia1	melanoma inhibitory activity 1	12587	1,68
Plac1	placental specific protein 1	56096	1,68
Cdh16	cadherin 16	12556	1,64
Cnksr2	connector enhancer of kinase suppressor of Ras 2	245684	1,64
Mthfd2l	methylenetetrahydrofolate dehydrogenase (NADP+ dependent) 2-like	665563	1,63
Pcgf1	polycomb group ring finger 1	69837	1,61
Pfn2	profilin 2	18645	1,61
Hspe1	heat shock protein 1 (chaperonin 10)	15528	1,58
Fmod	fibromodulin	14264	1,54
Cdh3	cadherin 3	12560	1,54
Maob	monoamine oxidase B	109731	1,54
Rpl23a	ribosomal protein L23a	268449	1,53
Flrt2	fibronectin leucine rich transmembrane protein 2	399558	1,53
Lgals12	lectin, galactose binding, soluble 12	56072	1,53
Nnat	neuronatin	18111	1,53
Rasef	RAS and EF hand domain containing	242505	1,53
Egfl6	EGF-like-domain, multiple 6	54156	1,53
Ctnnd2	catenin (cadherin associated protein), delta 2	18163	1,52
LOC674930	similar to suppressor of initiator codon mutations, related sequence 1	674930	1,5
Sox2	SRY-box containing gene 2	20674	1,57
Foxp2	forkhead box P2	114142	1,51

**Table 2:** Significant downregulated genes in the mycHIF3α expressing lungs.

Gene symbol	Gene name		Entrez ID	Fold Change	
Olfr767	olfactory receptor 767		258315	0,45	
Ass1	argininosuccinate synthetase 1		11898	0,53	
Pgam2	phosphoglycerate mutase 2		56012	0,56	
Gipr	gastric inhibitory polypeptide receptor		381853	0,58	
Olfr6	olfactory receptor 6		233670	0,6	
Igfbp6	insulin-like growth factor binding protein 6		16012	0,61	
Nppa	natriuretic peptide precursor type A		230899	0,61	
Dio3	deiodinase, iodothyronine type III		107585	0,63	
Mphosph6	M phase phosphoprotein 6		68533	0,64	
Plscr2	phospholipid scramblase 2		18828	0,64	
Ccin	calicin		442829	0,65	
Fabp5	fatty acid binding protein 5, epidermal		16592	0,65	
Nudcd3	NudC domain containing 3		209586	0,65	
Olfr171	olfactory receptor 171		258960	0,65	
Rtl1	retrotransposon-like 1		353326	0,66	
Rasgrf2	RAS protein-specific guanine nucleotide-releasing factor 2		19418	0,66	
Fabp12	fatty acid binding protein 12		75497	0,66	
Scnn1a	sodium channel, nonvoltage-gated 1 alpha		20276	0,66	
*Surfactant related genes*					
Scd1		stearoyl-Coenzyme A desaturase 1	20249		0,31
Sftpd		surfactant associated protein D	20390		0,65
*Clara cells marker*					
Scgb1a1(ccsp)		secretoglobin, family 1A, member 1 (uteroglobin)	22287		0,65
*Type I pneumocytes marker*					
Aqp5		aquaporin 5	11830		0,65

**Figure 7:**
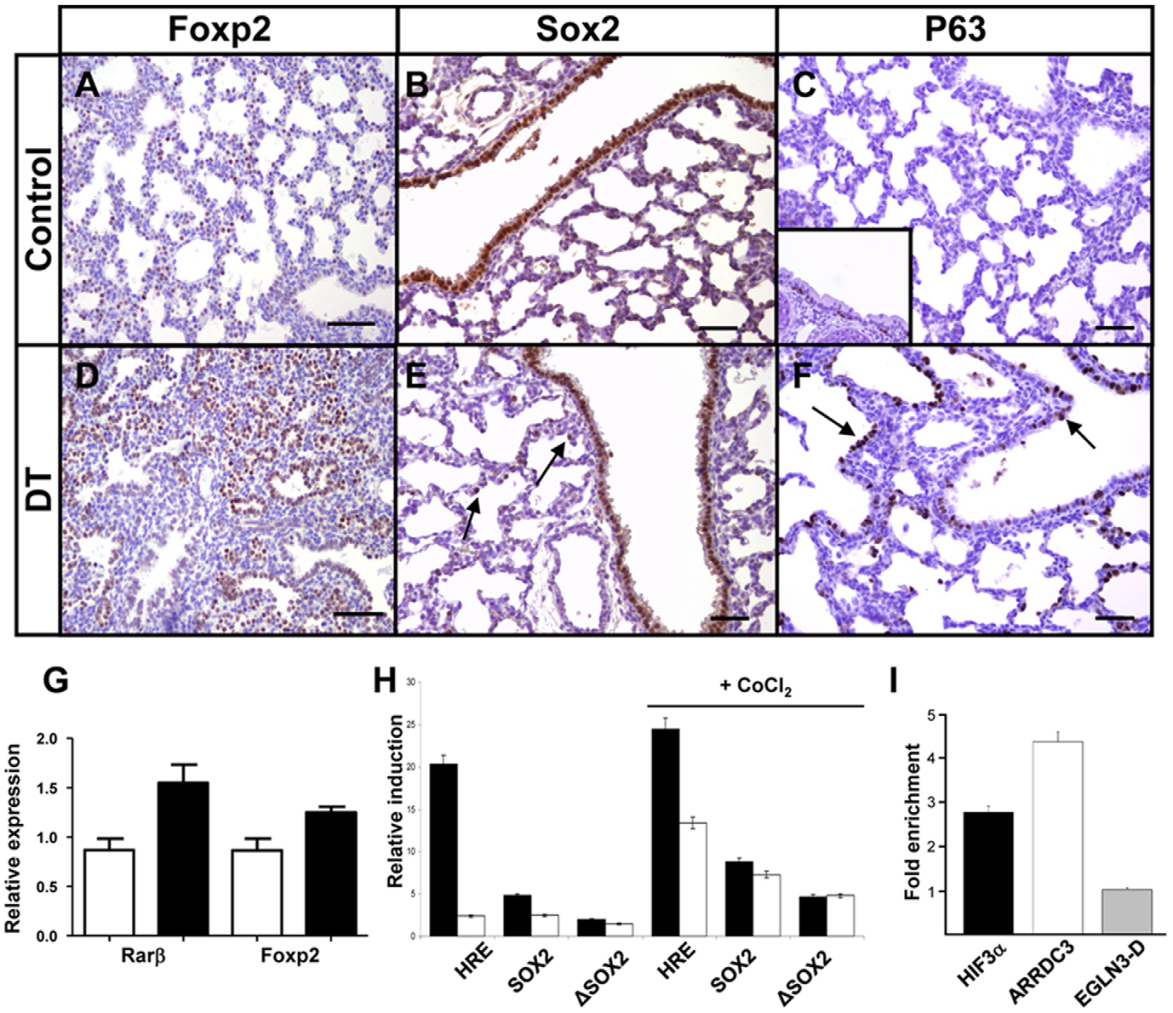
mycHIF3α induces the expression of proximal differentiation markers. mycHIF3α induces an expansion of the Foxp2 positive cells in the double transgenic lungs at gestational age E18.5 (A, D), as well as an expansion towards the distal parts of the lungs of Sox2 (B, E) and p63 (C, F). Sox2 was expressed in both proximal airways and alveolar epithelial cells in mycHIF3α transgenic lungs (*arrows*, E) at PN1. Basal cells are absent in control lungs (C), but are expressed in basal cells of trachea (C, insert). However, p63 is expressed in the proximal airways and alveolar epithelial cells in mycHIF3α transgenic lung (*arrows*, F). *Scale bar*: 200 µm (A and D) and 100 µm (B, C, E, F). (G) *Foxp2* and *Rarβ* are significantly upregulated in Hif3α transgenic lungs at gestational age E18.5 as shown by quantitative PCR. (Foxp2: 1.25 + 0.1 versus control 0.87 + 0.1, n = 3, P = 0.007; Rarβ: 1.55 + 0.1 versus control 0.87 + 0.1, n = 3, P = 0,009). White bars represent control lung samples, black bars represent mycHIF3α double transgenic lung samples. (H) Hif2α (black bars) and Hif3α (white bars) induce the 9*HRE-Luc (HRE) and Sox2-Luc (Sox2) as measured by the amount of luciferase activity. The fold induction of the HRE promoter is higher with Hif2α (20,3 fold and 24,5 fold under hypoxic conditions-CoCl_2_) than with Hif3α (2,4 fold and 13,4 fold under hypoxic conditions-CoCl_2_). The induction of the Sox2 promoter is higher with Hif2α than with Hif3α under normoxic conditions (4,8 versus 2,5), but equally strong under hypoxia mimicking conditions (8,8 versus 7,3). Data are presented as the induction (n-fold) relative to cells transfected with the corresponding reporter plasmid and control vector (pcDNA3). The values are the average of two duplicates, and standard deviations are: 0,04 (HRE-Hif2α), 0,02 (Sox2-Hif2α), 0,03 (ΔSox2-Hif2α), 0,08 (HRE-Hif3α), 0,24 (Sox2-Hif3α), 0,06 (ΔSox2-Hif3α), 0,53 (HRE-Hif2α+CoCl2), 0,007 (Sox2-Hif2α+CoCl2), 0,03 (ΔSox2-Hif2α+CoCl2), 0,88 (HRE-Hif3α+CoCl2), 0,02 (Sox2-Hif3α+CoCl2), 0,1 (ΔSox2-Hif3α+CoCl2). (I) Chromatin immunoprecipitation (ChIP) using anti-HIF3α antibody and chromatin isolated from A549 cells. Graph represents the fold enrichment of the HIF3α-specific binding to the conserved HRE of the SOX2 promoter compared to the IgG control ChIP. HIF3α also bound the ARRDC3 HRE region, and the enhancer region D of the EGLN3 gene served as negative control (EGLN3-D).

Among the upregulated genes are two transcription factors known to play important functions during lung development, *Foxp2* and *Sox2* [57], [64]. Foxp2 is important during lung development and is expressed in the distal parts of the lung. It represses the transcription of several distal cell markers, such as T1α, Spc, and Ccsp [65]. In our microarray analysis, *Foxp2* was significantly upregulated, which we validated by quantitative PCR (Table 1 and Figure 7G). Staining with a Foxp2 antibody show that the distribution of Foxp2 positive cells in Hif3α double transgenic lungs was expanded compared to control lungs (Figures 7A, D), suggesting that Hif3α suppressed the transcription of genes specific for alveolar epithelial cells through the induction of Foxp2. In addition, *Rarβ*, which is expressed at proximal sites in the lung from embryonic day 11 to 12 and not in the distal epithelium of the lung [66], [67], was significantly induced in Hif3α transgenic mice (Figure 7G), confirming the expansion of proximal cell makers in these lungs [64], [65].

Sox2 is important for pulmonary branching morphogenesis, epithelial cell differentiation and is exclusively expressed in the proximal parts of the lung [57]. However, in mycHIF3α expressing lungs, Sox2 is present in epithelial cells of both proximal airways and certain alveoli at postnatal day 1, suggesting that Hif3α is able to induce proximal cell fate (Figures 7B, E, arrows). The basal cell marker p63 is expressed in the esophagal and tracheal epithelium, and previously we showed that ectopic Sox2 expression induced the appearance of p63 positive cells in the epithelium of the bronchioles and enlarged distal airspaces [57]. Therefore, we analysed the distribution of basal cells in the mycHIF3α expressing lungs and found that p63 is abnormally expressed in the alveolar epithelial cells of mycHIF3α expressing lungs, contrasting the unique expression in the trachea (Figures 7C insert, *arrows* F). Collectively, our data indicate that mycHIF3α expression leads to the induction of crucial genes, such as Sox2, Foxp2 and Rarß, which cause airway epithelial cells to differentiate into proximal cell types.

### Hif3α binds the promoter region of Sox2 and induces transcription of Sox2

The promoter region of the *Sox2* gene contains two functional HREs, which are bound by Hif2α [68]. Since Sox2 is upregulated in Hif3α transgenic lungs, we analyzed whether Hif3α can directly induce the transcription of Sox2. Therefore, we first performed transcription reporter assays using a luciferase reporter construct under the influence of the Sox2 promoter containing two HREs, or two mutated HREs (Sox2-Luc and ΔSox2-Luc [68]). Hif3α induced the expression of the Sox2-Luc promoter about 2 fold, whereas the ΔSox2-Luc promoter was hardly induced compared to controls (Figure 7H). The positive control, HRE, was considerably induced by Hif2α, but only mildly by Hif3α, corresponding with the weak transcriptional activity of Hif3α [16], [42]. Under hypoxia-mimicking conditions, induced by adding CoCl_2_ to the medium, which inhibits prolyl hydroxylases by displacement of Fe(II) from their catalytic center [22], Hif3α could induce the 9*HRE-Luc considerably, and the difference with the Hif2α induced expression was much reduced (10 times versus 2 times). Moreover, the induction of the Sox2-Luc construct by Hif3α was 4 times higher than under normoxic conditions, and was comparable between Hif2α and Hif3α (Figure 7H). Subsequent analysis of the 1 kilobase region immediately upstream of the Sox2 transcriptional start site revealed that the most upstream of the two putative HRE sites was highly conserved between mice and human [68]. In order to investigate whether Hif3α could directly bind this conserved HRE site, we performed a chromatin immunoprecipitation of chromatin-protein complexes isolated from human A549 cells. Analysis of the HIF3α precipitated chromatin showed that the region containing the conserved HRE site in the SOX2 promoter region was indeed preferentially enriched compared to the IgG fraction (Figure 7I). ARRDC3 was used as a potential positive control, as it is bound by both HIF1α and HIF2α, and the enhancer region D of the EGLN3 gene served as negative control [36], [59]. Indeed, HIF3α did not bind to the EGLN3-D region, but did bind to the ARDDC3-HRE. This indicated that HIF3α could bind the HRE site present in the Sox2 promoter, suggesting a potential direct regulatory role of Hif3α in the transcription of Sox2.

So, Hif3α binds to the conserved HRE in the Sox2 promoter and weakly induces Sox2 expression, resulting in an abnormal Sox2 expression in airway epithelial cells of HIF3α transgenic lungs.

## Discussion

Hypoxia inducible factors are an important family of proteins involved in the regulation of the cellular response to hypoxia. Its functions are required from the earliest steps of mammalian life to the correct development of multiple organs and tissues, like the placenta, trophoblast formation, bone development, heart and vascular development (reviewed in [6], [8]). The importance of the hypoxia response was shown by the identification of human mutations in the VHL-HIF pathway in different diseases [9]. Gene ablation studies in mice have revealed in more detail the specific and important roles of the different subunits of the Hifα/Hifß heterodimers. Inactivation of the stable subunit, Hif1ß, resulted in severe embryonic defects and premature death [48], [49]. The disruption of the different Hifα genes identified specific roles for the individual Hifα isoforms. Hif1α knockout mice die early at gestation, have multiple developmental defects in neural tubeforrmation, vascularization, heart development, neural crest migration [69], [70], [71], whereas depending on the genetic background of the mouse strain, Hif2α knockout out mice ranging from early embryonic lethality to adulthood [51], [52], [53], [54].

### Hif genes and lung development

The lung is under continuous exposure of external oxygen and several (patho)-physiologic conditions trigger global or local hypoxia in the lung, resulting in pulmonary abnormalities to which HIFs contribute, such as lung cancer, acute lung injury and pulmonary hypertension (reviewed in [72]). Long term changes in oxygen levels, as experienced at high altitude gives rise to lung damage as a result of chronic mountain sickness. Recently, the *EPAS1* gene, encoding for HIF2α, was shown to be associated with adaptation of living at high altitude [73], [74], [75], [76].

Inactivation of Hif2α in mice resulted in respiratory distress and surfactant deficiency in newborns on a mixed genetic background [51]. Remarkably, heterozygous Hif1α^+/-^ or Hif2α^+/-^ mice showed a reduced increase in pulmonary arterial pressure and right ventricular hypertrophy upon exposure to chronic hypoxia in comparison with wild type mice [77], [78]. Ectopic expression of an oxygen-insensitive Hif1α transgene in lung epithelial cells during development resulted in defective branching, impaired epithelial maturation and respiratory distress. Moreover, increased expression of VegfA and VegfC was observed, leading to sub-pleural hemorrhaging [79]. We recently showed that the transgenic expression of an oxygen-insensitive mutant of Hif2α also lead to a late branching defects with enlarged alveoli and altered epithelial differentiation [55]. Contrasting the Hif1α transgenic study, we did not find increased levels of VegfA or endothelial abnormalities, even though the transgenes were expressed in the same manner. This indicates that Hif1α and Hif2α have different effects. In addition, the expression of Hif1α had not changed, whereas Hif3α expression was reduced in our Hif2α transgenic mice [55]. It seems that the effects of Hif1α are more widespread, whereas the number of affected genes by Hif2α is restricted, which is in line with previous reports describing target genes of Hif1α and Hif2α [35], [36], [80], [81], [82], [83], [84], [85].

The occurrence of the Hif3α isoforms is well described transcriptionally, but the functional analysis is complicated by the appearance of different splice variants [19], [26], [42], [43], [86]. Hif3α isoforms act as negative regulators of the traditional Hif1 (Hif1α/Hif1ß) and/or Hif2 (Hif2α/Hif1ß) driven hypoxia response by functioning as dominant negative modulators, effectively resulting in the transcriptional competition with Hif1 and Hif2 [16], [18], [26], [42], [43]. Gene ablation of Hif3α/NEPAS/IPAS, resulted in mice that were born alive with enlarged right ventricles and impaired lung remodelling [42]. Furthermore, they showed that expression of endothelin-1 is negatively influenced by Hif3α/NEPAS, by regulating the binding of Hif1α and Hif2α to the HRE sites if the ET-1 promoter, which may contribute to the observed phenotype. Remarkably, the expression of Vegf, a direct target of Hif1 and Hif2, had not changed, even though the expression of Hif1α and Hif2α was not affected. This hinted at a selective regulation of target genes by NEPAS/Hif3α during pulmonary development. Therefore, we conditionally expressed mycHIF3α in airway epithelial cells during embryonic development in order to further elucidate the role of Hif3α in pulmonary development.

### Cellular effects of mycHIF3α transgene expression

Since the NEPAS/Hif3α knockout mice suggested a selective regulation of genes by Hif3α, and our Hif2α transgenic mice showed a selective reduction in Hif3α expression, we conditionally expressed mycHIF3α in airway epithelial cells during embryonic development in order to further elucidate the role of Hif3α in pulmonary development. Analysis of mice expression a transgenic mycHIF3α in lung epithelium revealed a late branching morphogenesis defect with a reduced number of alveoli and changes in the differentiation of epithelial cell types.

Surprisingly, no apparent defects are observed early during lung development, even though the transgene is expressed. This may be due to the fact that at these stages of development, putative associating factors of Hif3α, like Hif2α and Hif2α, are not expressed yet. After the pseudoglandular stage of lung development, endogenous Hif2α becomes expressed in the cells positive for mycHIF3α and the effect of the mycHIF3α transgene starts to be noticeable. Histological analysis and gene expression profiling revealed changes in the differentiation of the developing pulmonary epithelium. We found reduced numbers of Clara cells, alveolar type I and type II cells, and in addition, basal cells were observed in atypical spatial positions. The expression pattern of diverse sets of genes was affected, and revealed that mycHIF3α expression mainly affects Hif2-directed transcription, although not all Hif2 target genes are equally affected. We show that expression of mycHIF3α in epithelial cells results in a down regulation of Hif2α, but not of Hif1α. This suggests that Hif3α is not a global regulator of the hypoxic response, but that Hif3α may selectively function to modulate Hif2α controlled target genes, supporting previous work [42]. The reduction in the expression level of Hif2α late in gestation may be due directly to the presence of mycHIF3α, or due to the impaired differentiation of the type II cells. However, it is clear that mycHIF3α does affect the differentiation of epithelial cells, and this could partly be explained by the aberrant activation of specific genes that are not part of the hypoxic response. Gene expression analysis does not show significant changes in typical hypoxia responsive genes, which indicates that Hif3α may have specific functions beyond the hypoxia response. Therefore, we provide first evidence for novel Hif3α functions beyond the hypoxia response.

The apparent increase in the mesenchymal compartment after the pseudoglandular stage does not seem to be induced by proliferation, as we did not observe an increase in mitotic cells in the mycHIF3α lungs. It may be due to either a delayed development of the double transgenic lungs, or, alternatively, to a specific response in epithelial cells triggered by mycHIF3α. Lysyl oxidase may be activated, which subsequently activates a cascade of proteins, such as Snail, involved in the repression of E-cadherin, and ultimately leading to epithelial-mesenchymal transition, as described for metastatic tumors [87], [88].

### Genes affected by mycHIF3α

The appearance of proximal cells at the expense of distal cells in the mycHIF3α lungs is paralleled by transcriptional changes in several genes, such as Sox2, Rarß and Foxp2. At this point, it remains to be seen whether all effects observed are directly related to mycHIF3α, or that the expression of mycHIF3α affects Hif1α and Hif2α specific complexes, thereby interfering with transcription of specific genes. The increased expression of mycHIF3α could lead to the formation of complexes that normally are not present in the cell, which would shift the balance between Hif2α and Hif3α.

We observed Sox2 positive cells at unusual sites in the lung, which was supported by the aberrant presence of p63 positive basal cells. Previously, we showed that Sox2 directly induces the appearance of basal cells [57]. Since a link was found between Hif2α and Sox2 transcription [68], we analyzed the putative regulation of the Sox2 gene by Hif3α. We show that Hif3α is capable of inducing basal expression of a reporter construct under the control of the Sox2 promoter containing two HRE sites. In addition, we show that HIF3α binds to the conserved HRE sequence in the Sox2 promoter, which suggests that Hif3α may contribute directly to the regulation of Sox2 expression. However, the minimal transcriptional activity of Hif3α, as also shown previously, may explain the appearance of only scattered Sox2 positive cells in the lungs of mycHIF3α mice [16], [26], [42]. In addition, depletion of individual HIFα genes by siRNA in human ES cells suggested that HIF3α upregulates HIF2α, which subsequently induced the expression of stem cell marker genes, like SOX2 [89]. Although this hypothesis is intriguing, no direct relationship was established, yet. It was also shown that ectopic expression of HIFs in cancer cell lines can induce embryonic stem cell markers, like SOX2 and NANOG [90]. The combination of weak transcriptional activity and the ability to act as a dominant negative modulator of Hif2α may be responsible for the transcriptional regulation of Sox2. These results directly show that through the expression of HIF3α, Sox2^+^ and p63^+^ basal cells appear and suggest that the balance between Hif2α and Hif3α may function as a modulator of basal cell emergence [68].

Besides the aberrant induction of Sox2 and p63, the expression domain of *Rarβ* was expanded distally in the mycHIF3α transgenic lungs. Rarβ knockout mice exhibited premature septation, and formed alveoli twice as fast as wild-type mice [66], [67], [91]. So, upregulation of *Rarβ* in mycHIF3α transgenic mice may in part explain the observed inhibition of pulmonary alveoli formation. We also detected an increase of Foxp2, which is a transcriptional repressor able to inhibit the expression of Ccsp and markers specific for distal epithelial cells, such as Spc and T1α [64], [65], [92]. Therefore, the reduced numbers of Clara cells (Ccsp^+^), alveolar type I (Aqp5^+^) and alveolar type II (Sftpd^+^) cells could be directly related to the upregulation of Foxp2. Recent findings showed that depletion of cells with CCSP promoter activity was associated with alveolar hypoplasia and respiratory failure, adding to the idea that Ccsp downregulation as a result of Hif3α-mediated Foxp2 upregulation, directly leads to reduced numbers of Clara cells [93].

The increase d expression of key genes in lung development, which lead to major changes in epithelial differentiation, was confirmed by the loss of expression of other cell type specific markers,, such as *Sftpd*, *Scd1* and *Abca3* for type II cells. At this point it is not clear if the reduced expression of the type II cell markers is the cause, or the result of the loss of type II cells. Previously, we showed a significant downregulation of *Scd1* and *Abca3* in Hif2α expressing transgenic mice, which suffered from respiratory distress and surfactant deficiency [55]. However, the mycHIF3α transgenic mice appeared to produce sufficient levels of Scd1 and Abca3 to support respiration, even though the expression of Hif2α is decreased.

Thus, the increased expression of Sox2, Rarß and Foxp2 in the developing mycHIF3α lungs may directly contribute to the cellular changes observed and explain the phenotypic abnormalities observed in these lungs. The effects may also be cell type specific, as increased HIF3α expression in vascular cells resulted in an antagonistic effect on hypoxia induced HIF1/HIF2 target genes [47].

### Concluding remarks

Although we cannot conclude that the dominant negative role of Hif3α as part of the hypoxic response is absent, our previous and current data do suggest that Hif2α and Hif3α have different target genes, during pulmonary development [55]. This is in line with previous findings describing common targets, as well as specific genes induced by Hif1α and Hif2α [80], [81], [82]. However, these studies used overexpression of Hif1α and Hif2α, which may cause aberrant complexes and loss of target gene specificity, as was reported for certain tumor cells [94]. Using siRNA and chromatin immuno-precipitation approaches, HIF1 and HIF2 target genes were identified [35], [36], [83], [84], [85]. Interestingly, it was shown that ETS transcription factors were involved in the regulation of HIF1 and HIF2 driven gene activation in MCF7 cells [83]. Knock down of ELK1 resulted in a reduction of hypoxia induced HIF2 dependent transcription. These data suggested a cooperation between ETS family members and HIF1 and HIF2 in the selection of target genes. An interesting idea is that target selection by HIFs may be cell specifically regulated by additional factors, adding to the complexity of the hypoxic response [8], [95]. This is also observed in the analysis of the different transgenic mouse models expressing Hif1α [79], and our studies with HIf2α or Hif3α, showing similarities and differences [55].

Thus, in spite of the limited functional significance of Hif3α/NEPAS in development as a global regulator of the hypoxia response, we demonstrate that Hif3α does contribute by balancing the function of the Hif regulated genes. Furthermore, Hif3α contributes to late branching morphogenesis, alveolar formation and epithelial differentiation. Moreover, the level of *Hif3α*, as well as Hif1α and Hif2α, is tightly regulated to ensure balance between the total number of proximal cells and distal cells.

## Supporting Information

Figure S1 **Expression of mycHIF3α leads to late branching defect.** External appearances of control (A and E) and mycHIF3α transgenic lungs (B and F) at E16.5 and E17.5 showed no apparent differences. Histological analysis of control (C and G) and mycHIF3α transgenic (D and H) lungs showed a gradual decrease in the number of air spaces and aberrant, late branching morphogenesis in mycHIF3α transgenic lungs. Anti-Myc epitope staining confirmed the expression of the mycHIF3α transgene in double transgenic lungs (D and H), which is absent in control lungs (C and G). Scale bars: 2 mm (A, B, E, F) or 200 µm (C, D, G, H).
(TIF)
