# Correction: Hypoxia Inducible Factor 3α Plays a Critical Role in Alveolarization and Distal Epithelial Cell Differentiation during Mouse Lung Development

**DOI:** 10.1371/journal.pone.0119359

**Published:** 2015-03-20

**Authors:** The PLOS ONE Staff

There is an error in Fig. 7. The authors have provided the corrected figure below.

**Fig 7:**
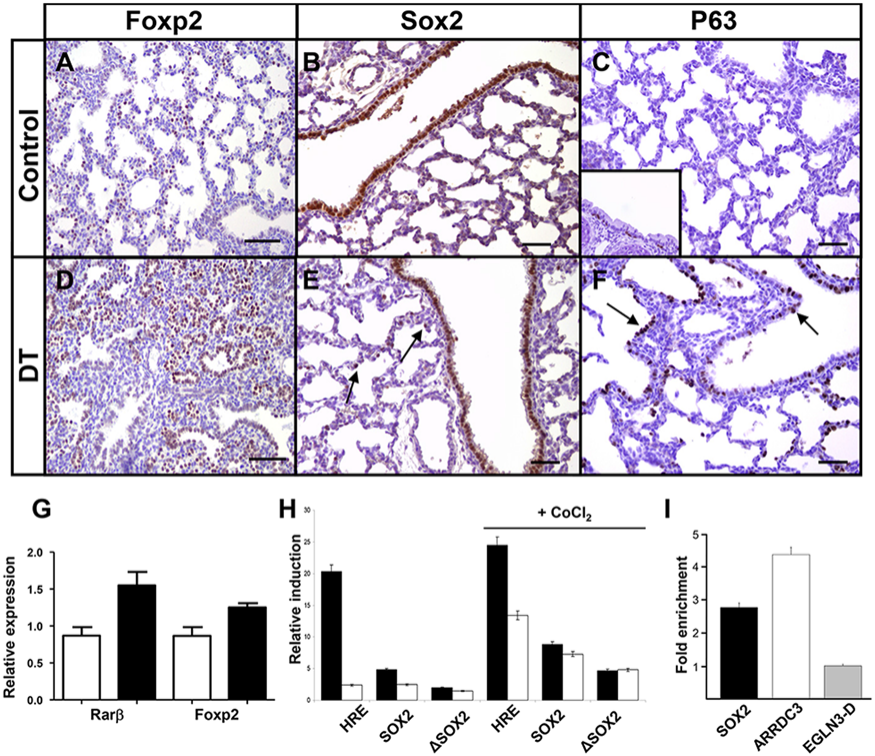
mycHIF3α induces the expression of proximal differentiation markers. mycHIF3α induces an expansion of the Foxp2 positive cells in the double transgenic lungs at gestational age E18.5 (A, D), as well as an expansion towards the distal parts of the lungs of Sox2 (B, E) and p63 (C, F). Sox2 was expressed in both proximal airways and alveolar epithelial cells in mycHIF3α transgenic lungs (arrows, E) at PN1. Basal cells are absent in control lungs (C), but are expressed in basal cells of trachea (C, insert). However, p63 is expressed in the proximal airways and alveolar epithelial cells in mycHIF3α transgenic lung (arrows, F). Scale bar: 200 μm (A and D) and 100 μm (B, C, E, F). (G) *Foxp2* and *Rarβ* are significantly upregulated in Hif3α transgenic lungs at gestational age E18.5 as shown by quantitative PCR. (Foxp2: 1.25 + 0.1 versus control 0.87 + 0.1, n = 3, P = 0.007; Rarβ: 1.55 + 0.1 versus control 0.87 + 0.1, n = 3, P = 0,009). White bars represent control lung samples, black bars represent mycHIF3α double transgenic lung samples. (H) Hif2α (black bars) and Hif3α (white bars) induce the 9*HRE-Luc (HRE) and Sox2-Luc (Sox2) as measured by the amount of luciferase activity. The fold induction of the HRE promoter is higher with Hif2α (20,3 fold and 24,5 fold under hypoxic conditions-CoCl_2_) than with Hif3α (2,4 fold and 13,4 fold under hypoxic conditions-CoCl_2_). The induction of the Sox2 promoter is higher with Hif2α than with Hif3α under normoxic conditions (4,8 versus 2,5), but equally strong under hypoxia mimicking conditions (8,8 versus 7,3). Data are presented as the induction (n-fold) relative to cells transfected with the corresponding reporter plasmid and control vector (pcDNA3). The values are the average of two duplicates, and standard deviations are: 0,04 (HRE-Hif2α), 0,02 (Sox2-Hif2α), 0,03 (ΔSox2-Hif2α), 0,08 (HRE-Hif3α), 0,24 (Sox2-Hif3α), 0,06 (ΔSox2-Hif3α), 0,53 (HRE-Hif2α+CoCl2), 0,007 (Sox2-Hif2α+CoCl2), 0,03 (ΔSox2-Hif2α+CoCl2), 0,88 (HRE-Hif3α+CoCl2), 0,02 (Sox2-Hif3α+CoCl2), 0,1 (ΔSox2-Hif3α+CoCl2). (I) Chromatin immunoprecipitation (ChIP) using anti-HIF3α antibody and chromatin isolated from A549 cells. Graph represents the fold enrichment of the HIF3α-specific binding to the conserved HRE of the SOX2 promoter compared to the IgG control ChIP. HIF3α also bound the ARRDC3 HRE region, and the enhancer region D of the EGLN3 gene served as negative control (EGLN3-D).
