# Supplementary figures and images for: Hypoxia Inducible Factor 3α Plays a Critical Role in Alveolarization and Distal Epithelial Cell Differentiation during Mouse Lung Development
Source: PLoS One. 2013 Feb 25;8(2):e57695. doi: 10.1371/journal.pone.0057695 (PMC3581546; doi:10.1371/journal.pone.0057695)

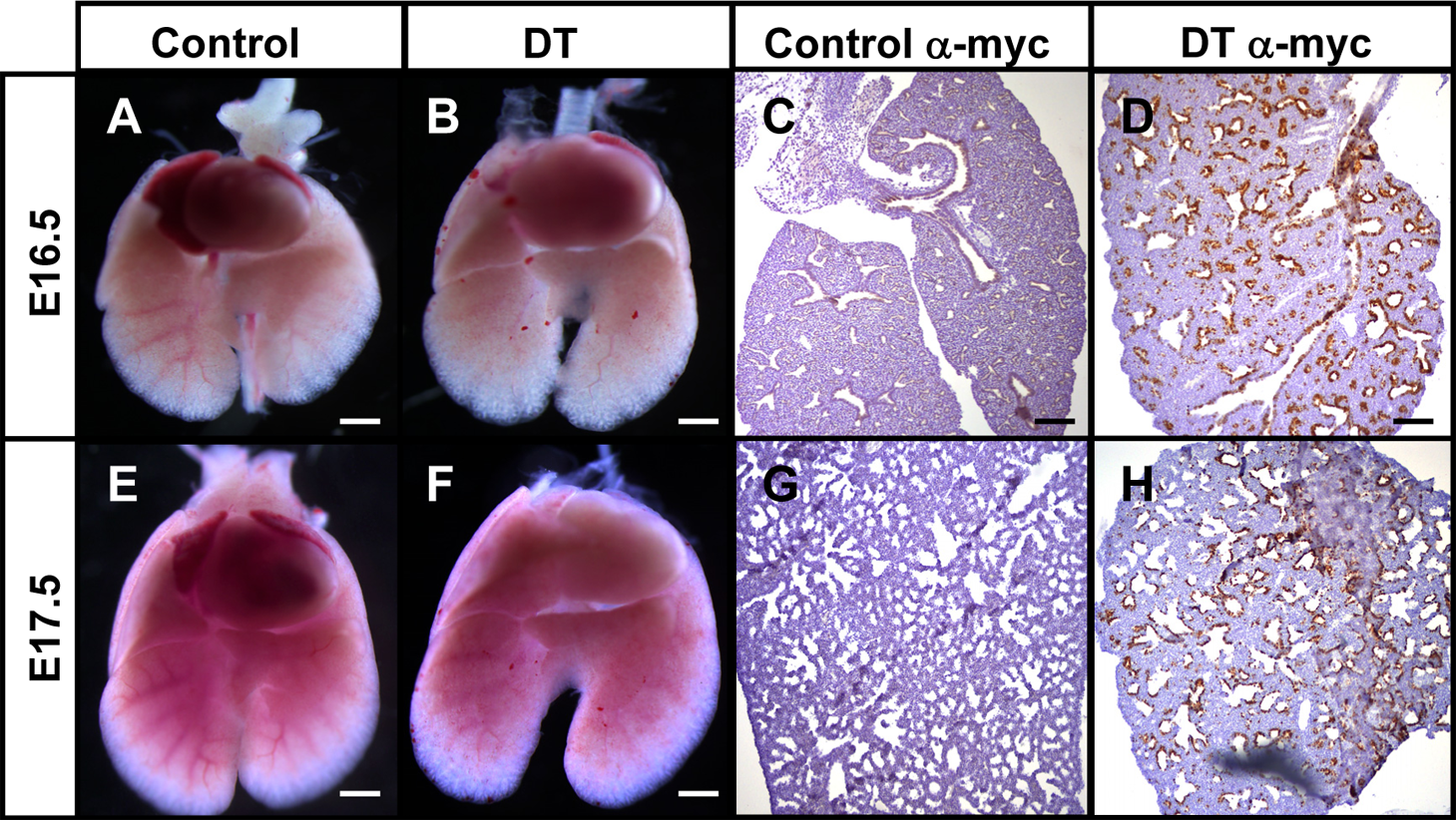

Supplement: Figure S1 — Expression of mycHIF3α leads to late branching defect. External appearances of control (A and E) and mycHIF3α transgenic lungs (B and F) at E16.5 and E17.5 showed no apparent differences. Histological analysis of control (C and G) and mycHIF3α transgenic (D and H) lungs showed a gradual decrease in the number of air spaces and aberrant, late branching morphogenesis in mycHIF3α transgenic lungs. Anti-Myc epitope staining confirmed the expression of the mycHIF3α transgene in double transgenic lungs (D and H), which is absent in control lungs (C and G). Scale bars: 2 mm (A, B, E, F) or 200 µm (C, D, G, H). (TIF) [file pone.0057695.s001.tif]
